# Dysglycemia in young women attenuates the protective effect against fatty liver disease

**DOI:** 10.3389/fendo.2022.971864

**Published:** 2022-11-21

**Authors:** Alejandra Pérez-Montes de Oca, María Teresa Julián, Guillem Pera, Llorenç Caballería, Rosa Morillas, Pere Torán, Carmen Expósito, Josep Franch-Nadal, Didac Mauricio, Nuria Alonso

**Affiliations:** ^1^ Department of Endocrinology and Nutrition, Hospital Germans Trias I Pujol, Barcelona, Spain; ^2^ Department of Medicine, Universitat Autònoma de Barcelona, Barcelona, Spain; ^3^ Unitat de Suport a la Recerca Metropolitana Nord, Fundació Institut Universitari per a la Recerca a l’Atenció Primària de Salut Jordi Gol i Gurina (IDIAPJGol), Mataró, Spain; ^4^ Centro d’Investigaciones Biomédicas en Red, Enfermedades Hepatologia y Digestivas, Barcelona, Spain; ^5^ Hepatology Department, Hospital Germans Trias I Pujol, Barcelona, Spain; ^6^ Center for Biomedical Research on Diabetes and Associated Metabolic Diseases (CIBERDEM), Instituto de Salud Carlos III, Madrid, Spain; ^7^ Primary Health Care Center Raval Sud, Gerència d’Atenció Primaria, Institut Català de la Salut, Barcelona, Spain; ^8^ Hospital de la Santa Creu i Sant Pau, Institut d’Investigació Biomèdica Sant Pau (IIB Sant Pau), Barcelona, Spain

**Keywords:** NAFLD, female sex, hyperglycemia, premenopausal woman, dysglycemia, fibrosis

## Abstract

**Introduction:**

Sexual dimorphism has been reported in non-alcoholic fatty liver disease (NAFLD), similar to the sex differences evident with cardiovascular disease. Type 2 diabetes mellitus (T2D) significantly increases the risk and severity of NAFLD, but there is scarce information on whether T2D or altered glucose metabolism can modify the prevalence of NAFLD in men and women of reproductive age.

**Purpose:**

To investigate the relationship between age, sex and NAFLD in subjects with and without dysglycemia.

**Materials and methods:**

We analyzed 2,790 patients. NAFLD was characterized using established diagnostic criteria: one or more positive results on the fatty liver index and hepatic ultrasound. Liver fibrosis (liver stiffness measurement [LSM] ≥8.0 kPa) was assessed by Fibroscan^®^. For analysis purposes, we included both T2D and prediabetes under the predefined condition of dysglycemia.

**Results:**

The global prevalence of NAFLD was higher in men than in women (50% and 34%; P<0.001), and the prevalence increased with age in both sexes. Older women (≥ 50 years) had a higher prevalence than younger women (<50 years), both in the overall cohort and in non-dysglycemic subjects. In dysglycemic subjects, the prevalence of NAFLD was slightly higher in men (68% vs 61%, p=0.021); in younger subjects, there were no differences in the prevalence of NAFLD between men and women (68% vs 64%, respectively; p=0.635). We found an interaction between dysglycemia and female sex (odds ratio [OR] 1.6 95% confidence interval [CI] 1.0-2.4, p=0.030), and between and age ≥50 years (OR 0.6, 95% CI 0.3-1.0, p=0.046). The global prevalence of LSM ≥8.0 kPa was higher in men compared with women (8% vs 4%; p< 0.001). This prevalence increased with age, mainly in men. We did not find any association between liver fibrosis and age and gender.

**Conclusions:**

While the global prevalence of NAFLD is higher in men than in women across all ages, younger women with dysglycemia have a similar risk of developing NAFLD as men of a similar age. Therefore, the presence of dysglycemia may erase the protective effect of female sex against fatty liver disease.

## Introduction

There is evidence that suggests that gender and reproductive status modulate the risk and severity of developing non-alcoholic fatty liver disease (NAFLD), similar to the sex differences that is evident with cardiovascular disease (1, 2). Data from several longitudinal studies reveal that the prevalence of NAFLD is higher in men than in women and that gender-specific differences exist in relation to age (3, 4). In women, menopausal status and older age are strong risk factors for NAFLD. Overall, the prevalence of NAFLD is higher in postmenopausal women when compared with premenopausal women, suggesting a protective anti-steatogenic effect of estrogens (5).

Regarding liver fibrosis, when it comes to sex differences, the results are mixed. In a previous cross-sectional population-based study, we described that male gender was a risk factor independently associated with increased liver stiffness measurement (LSM) assessed by transient elastography (TE) (6). In relation to the reproductive status, however, there is strong evidence that menopause increases the risk of liver fibrosis in the general population due to estrogen deficiency and dysmetabolic features that can occur in this reproductive period (2, 7).

On the other hand, it is well known that the presence of type 2 diabetes mellitus (T2D) significantly increases the risk and severity of NAFLD (8). Evidence from community-based studies reveals that the prevalence of increased LSM is also significantly higher in subjects with T2D compared with those without (9–11). Also, T2D and insulin resistance have been linked to increase the risk of advanced fibrosis in patients with nonalcoholic steatohepatitis (12). Data from a previous population study performed by our group revealed that the female sex was negatively associated with NAFLD in subjects with and without diabetes. However, only women without T2D were significantly negatively associated with having liver fibrosis (9).

Several epidemiological studies have shown that premenopausal women experience a higher degree of cardioprotection than men of similar age (1, 2). Moreover, evidence suggests that cardioprotection in women is lost under metabolic conditions, such as T2D (7, 8). To our knowledge, there is scarce information on whether T2D or impaired glucose metabolism can level or turn the prevalence of NAFLD in men and women of reproductive age. The aim of the present study was to investigate the relationship between age, reproductive status, and NAFLD in subjects with an alteration in glucose metabolism (dysglycemia: T2D or prediabetes) and in subjects without dysglycemia.

## Materials and methods

### Study design

This was a cross-sectional study in an urban area of Catalonia, in southwestern Europe. We included two well-characterized cohorts, both based on previous cross-sectional descriptive studies (9, 13). The two cohorts differed in their eligibility criteria and design; the first cohort was reflective of the general population in general practice in Catalonia and included subjects regardless of the presence of dysglycemia, while the second cohort included subjects with T2D. In the first cohort, the recruitment was conducted in several municipalities in the northern part of the Barcelona metropolitan area between April 2012 and January 2016. Participants in the study were randomly selected from a total of 162,950 subjects aged 18 to 75 years from the registries of the primary health care centers of the municipalities included in the study. Patients with a current history of liver disease, including cholestasis, hepatitis C or B virus infection, and high-risk alcohol consumption were excluded from the study. Other exclusion criteria were active malignancy, other severe diseases (congestive heart failure New York Heart Association >2, chronic obstructive pulmonary disease Global Initiative for Chronic Obstructive Lung Disease >2, chronic kidney disease requiring dialysis, previous organ transplantation, and severe neurologic diseases), or admission to long-term nursing homes. The second cohort was recruited from an outpatient clinic in Lleida, also in Catalonia, Spain. This was also a population-based cohort recruited to evaluate the prevalence of prediabetes in the general population (13). The inclusion criteria were age range 40–75 years; T2D diagnosis; absence of established impaired renal function (calculated glomerular filtration rate (eGFR) <60 ml/min) and absence of known cardiovascular disease.

### Definitions

Aspartate transaminase (AST) and/or alanine transaminase (ALT) values >40 IU/L were considered as high serum transaminases. To determine high-risk alcohol consumption, standard drinking units/week were used: 21 for men and 14 for women. In the absence of secondary causes of steatosis, NAFLD was characterized using established diagnostic criteria: one or more positive results on the fatty liver index (FLI) and abdominal echography. The FLI uses body mass index (BMI), waist circumference, serum gamma-glutamyltransferase (GGT), and triglycerides to calculate the amount of fat in the liver. A FLI score of 60 or higher was used to define NAFLD (6, 14, 15).

Liver fibrosis was measured by TE, performed by three specially trained liver nurses. TE was conducted using the Fibroscan^®^ system (402, Echosens^®^, Paris, France) using only the M probe (the XL probe was unavailable). Moderate-to-advanced liver fibrosis was defined as an increased LSM ≥8.0 kPa. In a recent large population-based investigation, this cut-off demonstrated the prediction of substantial liver fibrosis, particularly in people with NAFLD (10). The LSM by TE was only available in the first cohort.

T2D and prediabetes diagnosis were based on a registered diagnosis in the clinical records or having an HbA1c ≥6.5% or fasting glucose ≥126 mg/dL and Hb1Ac 5.7-6.4% or fasting glucose 100-125 mg/dL, respectively (16). Abdominal obesity was defined as waist circumference ≥102 cm in men or ≥88 cm in women. For the purpose of this analysis we included both T2D and prediabetes subjects under the predefined category of dysglycemia. Age ≥50 years old was considered to define older subjects and age < 50 years old was used to define younger subjects according to a previous review (2).

### Statistical analysis

Continuous variables are expressed as mean ± standard deviation (SD) and categorical variables as frequency and percentage. Comparison of proportions and prevalences were tested using chi-squared tests. Univariate logistic regression models were performed to assess the relationship between NAFLD or fibrosis (outcomes) and gender, age over 50 and dysglycemia. Additionally, the same model was applied using a computed variable integrating the combination of gender, age and dysglycemia to better show how each profile is more or less associated with NAFLD. Finally, logistic regression models including gender, diabetes, age over 50, abdominal obesity and the interaction terms between the first 3 variables were performed. All the analyses were performed using Stata v17.

### Ethics

All participants gave their written informed consent. The protocol followed the current and relevant standards and regulations, and it was authorized by the Fundació Gol I Gorina’s Ethics Committee (P11/58) (Barcelona, Spain) and the Ethics Committee of Hospital Arnau de Vilanova (Lleida, Spain).

## Results

### Characteristics of the study population

**Figure 1:**
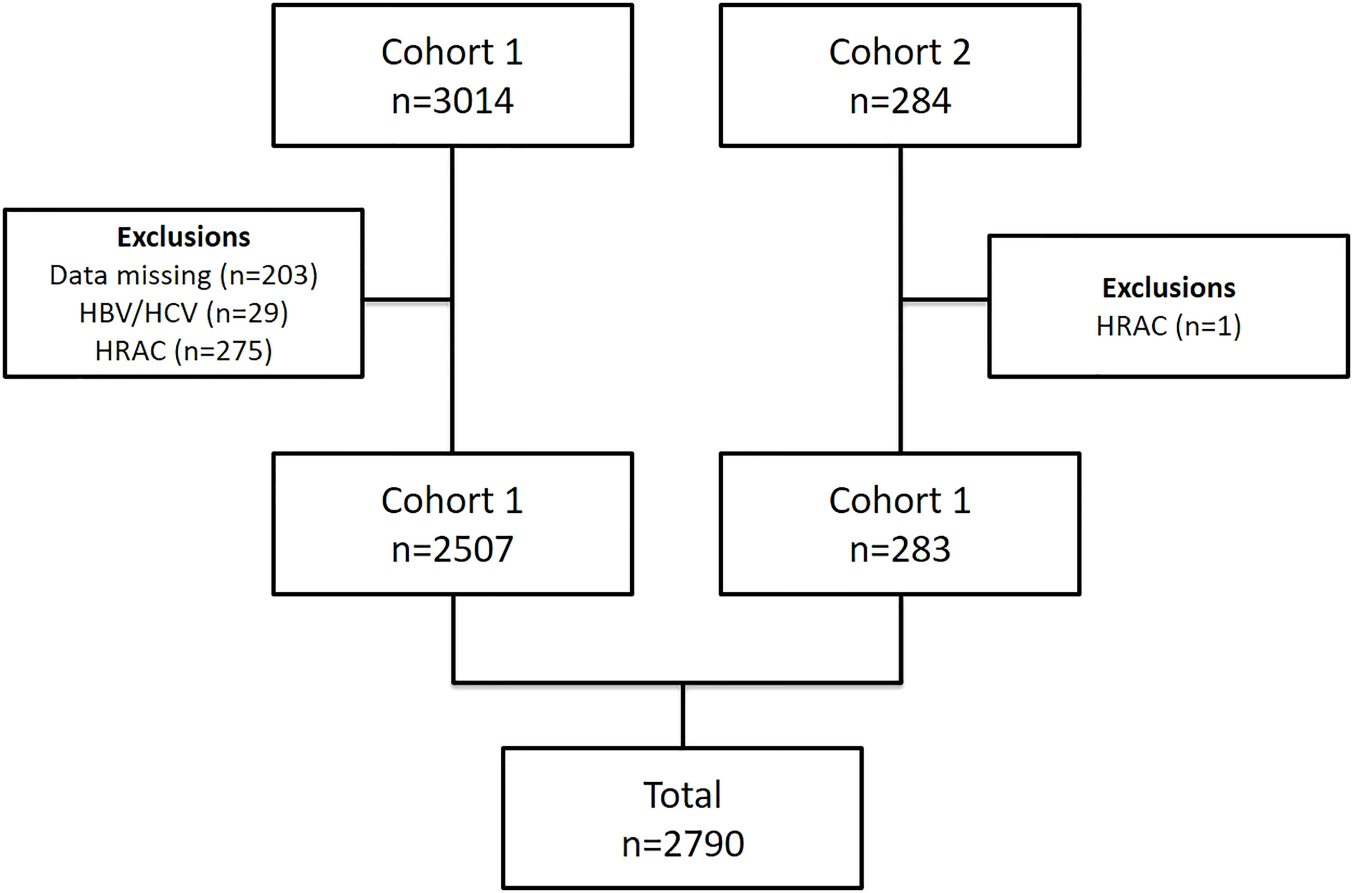
Flowchart of study selection. HBV, Hepatitis B Virus, HCV, Hepatitis C Virus; HRAC, high-risk alcohol consumption.

We analyzed a total of 2,790 subjects, 2,507 from the first cohort and 283 from the second cohort (**Figure 1**). The median age was 55 ± 12 years old, 60% were female with a mean BMI of 29 ± 5 kg/m^2^. A third of the patients were dysglycemic and 41% had NAFLD. The main characteristics of the subjects are represented in **Table 1**.

**Table 1:** Baseline characteristics of study population.

	Cohort 1	Cohort 2	Total
Age (n,SD)	55 ± 12	59 ± 10	55 ± 12
≥ 50 years old n(%)	1693 (68)	224 (79)	1917 (69)
Women (n,%)	1530 (61)	145 (51)	1675 (60)
BMI (Kg/m^2^) (n,SD)	28 ± 5	32 ± 6	29 ± 5
Abdominal obesity (n,%)	1262 (50)	226 (80)	1488 (53)
Non-smokers (n,%)	1249 (50)	138 (49)	1387 (50)
Ex-smokers (n,%)	706 (28)	84 (30)	790 (28)
Smokers (n,%)	540 (22)	59 (21)	599 (22)
NAFLD (n,%)	942 (38)	190 (67)	1132 (41)
T2D (n,%)	297 (12)	283 (100)	580 (21)
T2D /pre-diabetic (n,%)	606 (24)	283 (100)	889 (32)
Fasting glucose (mg/dL,SD)	100 ± 26	159 ± 56	106 ± 35
HbA_1c_ (%,SD)	5.7 ± 0.7	7.8 ± 1.4	5.9 ± 1.0
Total cholesterol (mg/dL,SD)	212 ± 39	189 ± 36	210 ± 39
LDL-cholesterol (mg/dL,SD)	134 ± 34	111 ± 31	132 ± 35
HDL-cholesterol (mg/dL,SD)	55 ± 13	51 ± 14	55 ± 13
Cholesterol remanents	24 ± 13	27 ± 16	24 ± 14
Non HDL-cholesterol (mg/dL,SD)	157 ± 37	138 ± 35	155 ± 37
Triglycerides (mg/dL,SD)	121 ± 73	141 ± 98	123 ± 76
Atherogenic dyslipidemia (n, %)	255 ± 10	53 ± 19	308 ± 11
AST and/or ALT >40 (n, %)	207 (8)	46 (16)	253 (9)
Fibroscan (kPs, SD)	4.9 ± 2.1		
Fibroscan ≥8 (n,%)	139 (6)		

SD, standard deviation; NAFLD, non-alcoholic fatty liver disease; T2D, Type 2 diabetes; HbA1c, glycated hemoglobin; AST,aspartate aminotransferase; ALT,alanine aminotransferase

### Prevalence of NAFLD according to age and sex

The global prevalence of NAFLD was 50% for men and 34% for women (p< 0.001). According to age, this prevalence was lower in younger subjects (38% in men and 19% in women), and higher in older subjects (56% in men and 41% in women). In all age groups, the prevalence was higher in men than in women (p<0.001). The overall prevalence of NAFLD according to age and sex is shown in **Table 2**.

**Table 2:** Prevalence of NAFLD according to age and sex.

	Global			<50 years old			≥50 years old	
	Prevalence (%)	p		Prevalence (%)	P		Prevalence (%)	p
Global								
Men (n=1115)	50	<0.001	Men (n=364)	38	<0.001	Men (n=751)	56	<0.001
Women (n=1675)	34		Women (n=509)	19		Women (n=1166)	41	
Dysglycemia								
Men (n=432)	68	0.021	Men (n=75)	68	0.635	Men (n=357)	68	0.024
Women (n=457)	61		Women (n=47)	64		Women (n=410)	60	
Non-dysglycemia								
Men (n=683)	39	<0.001	Men (n=289)	30	<0.001	Men (n=394)	46	<0.001
Women (n=1218)	24		Women (n=462)	14		Women (n=756)	30	

NAFLD, Non-alcoholic fatty liver disease.

In subjects with normoglycemia, the prevalence of NAFLD was significantly higher in men than in women, in line with the finding in the general cohort (39% and 24%; p<0.001). Overall, men had more risk than women despite their age, and older women had twice the prevalence than the younger ones (30% vs. 14%, p<0.001). Focusing on subjects with dysglycemia, however, the global prevalence of NAFLD was more similar between sexes, being only slightly higher in men than in women (68% vs 61%, p=0.021). Similar results were found in older subjects (68% men vs 60% women; p=0.024). When we analyzed the sex differences in younger subjects, there were no significant differences in the prevalence of NAFLD between men and women (68% vs 64%; p=0.635). Also, younger dysglycemic women had a similar prevalence than older women with dysglycemia, as well as to males regardless of age. Among women, in young dysglycemic women, the prevalence of NAFLD was 64% vs 14% (p<0.001) in normoglycemic ones, while in older women this tendency remained but was more attenuated (60% vs. 30%, respectively, p<0.001).

### Regression model for NAFLD, age and sex

We applied a logistic model to investigate the association of age, sex and dysglycemia with NAFLD. The univariate model found that female sex was a protective factor (odds ratio [OR] 0.5; p<0.001), whereas age ≥50 years (OR 2.4; p<0.001) and dysglycemia (OR 4.3; p<0.001) were associated with NAFLD. Taking as reference normoglycemic young women, a young dysglyemic woman had ten times higher risk of NAFLD (p<0.001), a slightly higher risk when compared to older hyperglycemic women, and an almost similar risk when compared to all men with dysglicemia in the cohort. The complete univariate model is shown in **Table 3**.

**Table 3:** Univariate model for NAFLD in our general cohort.

	OR	95% Cl	P	n
Woman	0.5	0.4-0.6	<0.001	554
≥50 years	2.4	2-2.9	<0.001	234
Dysglycemia	4.3	3.6-5.1	<0.001	318
Non-dysglycemic young men	2.6	1.8-3.7	<0.001	87
Dysglycemic young men	12.8	7.4-22.1	<0.001	24
Non-dysglycemic old men	5	3.6-7	<0.001	180
Dysglycemic old men	12.8	9.1-18	<0.001	114
Non-dysglycemic young women	Ref			66
Dysglycemic young women	10.6	5.5-20.3	<0.001	17
Non-dysglycemic old women	2.6	1.9-3.5	<0.001	228
Dysglycemic old women	9.1	6.6-12.6	<0.001	163

NAFLD, non-alcoholic fatty liver disease; young, <50 years old; old, ≥50 years old.

After adjusting for abdominal obesity and including interactions, the presence of dysglycemia (OR 3.1; p<0.001), abdominal obesity (OR 28, p<0.001), and age over 50 years (OR 1.6; p<0.001) were factors positively associated with NAFLD, whereas female sex was negatively associated with this condition (OR 0.1; p<0.001). We found two interactions: between dysglycemia and female sex (OR 1.6 95% confidence interval [CI] 1.0-2.4, p=0.030) and between dysglycemia and age over 50 years (OR 0.6, 95% CI 0.3-1.0, p=0.046). No interaction between gender and age was found.

### Prevalence of moderate-to-advanced liver fibrosis according to age and sex

Subsequently, we analyzed the prevalence of liver fibrosis assessed by TE. Data from TE was only available for the first cohort (n= 2507). The prevalence of moderate-to-advanced liver fibrosis in the general cohort defined by LSM ≥8.0 kPa, was higher in men compared to women (8% vs 4%; p< 0.001). This prevalence increased with age, mainly in men (4% in young men vs 11% in older men). Among young subjects, no differences were observed in the prevalence of LSM ≥8.0 kPa regarding sex. Similar findings were found in normoglycemic subjects. The prevalence of liver fibrosis according to age and sex is presented in **Table 4**.

**Table 4:** Prevalence of moderate-to-advanced liver fibrosis according to age and sex.

	Global			<50 years old			≥50 years old	
	Prevalence (%)	p		Prevalence	P		Prevalence (%)	P
**Global**								
Men (n=977)	8	<0.001	Men (n=327)	4	0.417	Men (n=650)	11	<0.001
Women (n=1530)	4		Women (n=487)	3		Women (n=1043)	4	
**Dysglycemia**								
Men (n=294)	18	0.012	Men (n=38)	11	0.348	Men (n=256)	19	0.013
Women (n=312)	11		Women (n=25)	4		Women (n=287)	11	
**Non-dysglycemia**								
Men (n=683)	5	0.001	Men (n=289)	3	0.888	Men (n=394)	6	<0.001
Women (n=1218)	2		Women (n=462)	12		Women (n=756)	1	

Moderate-to-advanced liver fibrosis was defined by transient elastography ≥8 Kpa.

The prevalence of moderate-to-advanced liver fibrosis was higher in subjects in the dysglycemic group than in subjects without dysglycemia regardless of their age and sex. In general, men with dysglycemia had a higher prevalence of liver fibrosis than dysglycemic women (18% vs 11%; p=0.012), mainly in older subjects. However, this sex difference disappeared when younger subjects were analyzed (11% vs 4%; p= 0.348).

The logistic regression model revealed that older and younger men with dysglycemia had a 8.7-fold and 4.4-fold risk of having moderate-to-advanced hepatic fibrosis compared to young normoglycemic women. The difference in the risk of having liver fibrosis in younger hyperglycemic women compared to younger normoglycemic ones did not reach statistical significance. In the multivariate analysis adjusted for abdominal obesity (OR 5.9; p<0.001), having dysglucemia was associated with moderate-to-advanced liver fibrosis (OR 3.8; p<0.001). Female sex was a protective factor (OR 0.3; p<0001). No interactions were found to be significant.

## Discussion

There is little information on the impact of glucose metabolism on NAFLD according to sex and age. Most of the studies evaluated either diabetes or reproductive status but not both concomitantly. To our knowledge, the present study describes for the first time the influence of dysglycemia on the prevalence of NAFLD and liver fibrosis assessed by TE and its relationship with age and sex.

The present study found that the prevalence of NAFLD was higher in men compared to women in the whole cohort (50% vs 34%), independent of age. It is known that NAFLD has a higher prevalence among men than women (3, 4, 17). A study reported by Wong et al. found that NAFLD, assessed by proton magnetic resonance spectroscopy, was more prevalent in men than in women (37% vs. 23%). The prevalence of fatty liver (FL) was especially low in women below age 50 years (12-16%), but it increased steadily after menopause, while in men it remained constant across ages (17). According to this study, we found that older women had double the prevalence of NAFLD than younger women but not more than older men. This result is consistent with other studies (18, 19). Moreover, in the Framingham Heart Study that was designed to evaluate the incidence of hepatic steatosis in a community based-cohort study of 685 patients, the frequency of FL measured by computed tomography was similar in postmenopausal women and men (19% vs. 22%), while it was lower in premenopausal women (9%) (20). All these findings suggest that men and postmenopausal women are more likely to have NAFLD. The exact reason is unknown, but dysregulated sex hormones, especially estrogens, appear to have an important role due to their various effects on the liver (17). Given that estradiol has an inhibitory effect on inflammatory markers such as TGF-β1, TNF-α, IL-6, and IL-1B, it is hypothesized that the fertility status may have a protective influence on the development of NAFLD (5). Regarding NAFLD biomarkers, there is evidence on the limitation and necessity to confirm the value of transaminases in the clinical diagnosis of NAFLD. A meta-analysis found that normal ALT values are present even in 25% of the patients with NAFLD and 19% with nonalcoholic steatohepatitis (NASH). In our study, only 9% of the patients had elevated ALT and/or AST (21).

It is well known that diabetes plays an important role in the incidence and progression of NAFLD. The prevalence of advanced fibrosis in patients with NAFLD and T2D was estimated to be 17% in a previous meta-analysis study (22). In a recent study published by our group in a general population, the prevalence of NAFLD was higher in subjects with T2D compared to those without (9). Moreover, not only T2D but insulin resistance have shown to play an important role in the progression of NAFLD, specifically an increased risk of advanced fibrosis in patients with NASH (12). This occurred even when subjects are not obese (23). According to recent research, the distribution of adipose tissue may be more important for the development of metabolic diseases than the actual amount of body fat. There may be a connection between the amount of abdominal adipose tissue and obesity-related NAFLD (24). In the present study, the presence of abdominal adiposity was the strongest risk for NAFLD and hyperglycemia was a risk factor for NAFLD and for moderate-to-advanced liver fibrosis in multivariate analysis. On the other hand, there is evidence that the development of T2D causes a disproportionately increased risk of CVD in women, approaching that of men (19). In addition, the presence of gender and age dimorphism has been reported in NAFLD. However, if this phenomenon occurs in the presence of diabetes is not understood.

As expected, among hyperglycemic subjects, the prevalence of NAFLD was higher compared to non-hyperglycemic subjects, and it was slightly higher in men. If we focus on sex differences according to age, we did not find significant differences in the prevalence of NAFLD between younger men and younger women with dysglycemia. Hereby, we report for the first time that younger women lose the protective effect against liver steatosis in the presence of dysglycemia. In fact, a younger dysglycemic woman had ten times the risk of harboring NAFLD compared to a young non-hyperglycemic woman, a risk that was similar to all men in the global cohort. A recent study from the UK examined sex differences in intra-organ fat and hepatic VLDL1-triacylglycerol export before and after major dietary weight loss and found that in T2D, women have liver and pancreas fat levels as high as those of men, associated with raised hepatic VLDL1-TG production rates. They concluded that these changes may contribute to women with diabetes having a disproportionately higher cardiovascular risk (25).

In relation to liver fibrosis, there are inconclusive data on the presence of age-specific sex differences (19). In a prospective cohort study of 40,700 adults with NAFLD, obesity and weight gain, but not sex, were related to the risk of fibrosis progression measured by the AST to Platelet Ratio Index (APRI) (26). In a previous cross-sectional population-based study, we described that one of the factors independently associated with increased LSM was male gender (6). Female sex was negatively associated with moderate-to-severe liver fibrosis but this did not reach a statistical difference in subjects with diabetes (9). Finally, in another study, the occurrence of advanced fibrosis was substantially higher in postmenopausal women (27.6%) compared to premenopausal women (14.4%) and men (22.2%) in 541 persons with biopsy-proven NAFLD. After adjusting for several metabolic factors, postmenopausal women had a 1.5-fold increased risk of having more severe liver fibrosis than premenopausal women (7). In the present study, we found that the prevalence of liver fibrosis increased with age and was two-fold higher in men than in women, as also described in other studies (6). However, sex differences were attenuated in younger subjects regardless of the presence or absence of hyperglycemia. As expected, when dysglycemic subjects were evaluated, the prevalence of moderate-to-advanced liver fibrosis was higher than in non-hyperglycemic subjects. In a population based-study reported by our group, the prevalence of liver fibrosis assessed by TE was higher in the diabetic population compared to those without (21% vs 3%). In general, we found that hyperglycemic men had a higher prevalence of liver fibrosis compared to women, mainly in older subjects. In contrast to what occurs in NAFLD, we did not find an association between liver fibrosis and reproductive status (i.e between pre-menopausal and post-menopausal women). However, these results must be interpreted with caution due to the small size of the sample.

Our study had some limitations that deserve mentioning: (i) Fibroscan was only performed on one of the two cohorts, so the number of subjects with dysglycemia who had LSM was relatively small. This fact may have affected the statistical power, and consequently the liver fibrosis results; (ii) the exact age of onset of menopause is unknown in these cohorts. Age ≥50 years old was considered to define older subjects and age <50 years old was used to define younger subjects according to a previous review (2); (iii) The XL probe was not available in this study; the percentage of unreliable measurements of liver stiffness was only 1.5% as the study subjects were not overtly obese, with few patients having class 2 and 3 obesity. The use of an XL probe could reduce this failure rate, although we do not believe it would have impacted the main results of the study”.; (iv) we don’t have available the data of the specific antidiabetic treatment received by each individual that could influence the progression of NAFLD.

## Conclusions

We hereby report for the first time that, similar to what occurs in cardiovascular disease; dysglycemia may eliminate the protective effect of female sex on NAFLD. In a hyperglycemic scenario, being a woman at a younger age had a negative impact on the prevalence of NAFLD. An active search for hepatic steatosis would be recommended in younger women with any degree of dysglycemia.

## Data availability statement

The original contributions presented in the study are included in the article/Supplementary Materials. Further inquiries can be directed to the corresponding authors.

## Ethics statement

The studies involving human participants were reviewed and approved by Fundació Gol I Gorina’s Ethics Committee (P11/58) (Barcelona, Spain) and the Ethics Committee of Hospital Arnau de Vilanova (Lleida, Spain). The patients/participants provided their written informed consent to participate in this study.

## Author contributions

All authors had full access to all of the data in this study and take complete responsibility for the integrity of the data and accuracy of the data analysis. All named authors meet the International Committee of Medical Journal Editors (ICMJE) criteria for authorship for this manuscript, take responsibility for the integrity of the work as a whole, and have given final approval for the version to be published.

## Funding

The project received a research grant from the Carlos III Institute of Health, Ministry of Economy and Competitiveness (Spain), awarded on the 2011 call under the Health Strategy Action 2013–2016, within the National Research Program oriented to Societal Challenges, within the Technical, Scientific and Innovation Research National Plan 2013–2016, with reference PI11/0267, PI14/00407, co-funded by European Union European Regional Development Fund funds. Also supported by grants from Fondo de Investigación Sanitaria Instituto de Salud Carlos III-Subdirección General de Evaluación and the European Regional Development Fund Fondo Europeo de Desarrollo Regional (PI16/00043), Carlos III Institute of Health (PI21/00817), the Agencia de Gestió d’Ajuts Universitaris i de Recerca, the European Horizon 20/20 program, H20/20-SC1-2016- RTD, Institució Catalana de Recerca I Estudis Avançats Academy Award (P G).

## Conflict of interest

The authors declare that the research was conducted in the absence of any commercial or financial relationships that could be construed as a potential conflict of interest.

## Publisher’s note

All claims expressed in this article are solely those of the authors and do not necessarily represent those of their affiliated organizations, or those of the publisher, the editors and the reviewers. Any product that may be evaluated in this article, or claim that may be made by its manufacturer, is not guaranteed or endorsed by the publisher.
